# A Text-Book of Bacteriology

**Published:** 1902-02

**Authors:** 


A Text-Book of Bacteriology. By George M. Sternberg, M. D., LL. D., Surgeon-General U. S. Army; Ex-President of the American Medical Association and of the American Public Health Association, etc. Octavo, pp. 718. Illustrated by heliotype and chromo-lithographic plates and 200 engravings. Second revised edition. New York: William Wood & Co. 1901. [Price, cloth, $4.50 net; brown sheep, $5.25 net.]

The above text-book, previously a manual of bacteriology, is the representative American work on bacteriology. The pleasing feature of colored illustrations and original photomicrographs reproduced by heliotype, has been retained. The chapters on toxins and toxalbumins, susceptibility, immunity and protective inoculation, have all been brought up to date and are excellent. A chapter on bacterial diseases of plants has been added and is of interest. The book forms an excellent laboratory text and reference book and is without doubt the best work of its kind in the English language.—H.R.G.

